# Kahweol, a natural diterpene from coffee, induces peripheral antinociception by endocannabinoid system activation

**DOI:** 10.1590/1414-431X2021e11071

**Published:** 2021-10-29

**Authors:** L.S. Guzzo, C.C. Oliveira, R.C.M. Ferreira, D.P.D. Machado, M.G.M. Castor, A.C. Perez, F. Piscitelli, V. Di Marzo, T.R.L. Romero, I.D.G. Duarte

**Affiliations:** 1 Departamento de Farmacologia, Instituto de Ciências Biológicas, Universidade Federal de Minas Gerais, Belo Horizonte, MG, Brasil; 2 Endocannabinoid Research Group, Institute of Biomolecular Chemistry, National Research Council, Pozzuoli, Napoli, Italy

**Keywords:** Kahweol, Coffee, Antinociception, Cannabinoid system, Endocannabinoid

## Abstract

Kahweol is a compound derived from coffee with reported antinociceptive effects. Based on the few reports that exist in the literature regarding the mechanisms involved in kahweol-induced peripheral antinociceptive action, this study proposed to investigate the contribution of the endocannabinoid system to the peripheral antinociception induced in rats by kahweol. Hyperalgesia was induced by intraplantar injection of prostaglandin E_2_(PGE_2_) and was measured with the paw pressure test. Kahweol and the drugs to test the cannabinoid system were administered locally into the right hind paw. The endocannabinoids were purified by open-bed chromatography on silica and measured by LC-MS. Kahweol (80 µg/paw) induced peripheral antinociception against PGE_2_-induced hyperalgesia. This effect was reversed by the intraplantar injection of the CB_1_ cannabinoid receptor antagonist AM251 (20, 40, and 80 μg/paw), but not by the CB_2_ cannabinoid receptor antagonist AM630 (100 μg/paw). Treatment with the endocannabinoid reuptake inhibitor VDM11 (2.5 μg/paw) intensified the peripheral antinociceptive effect induced by low-dose kahweol (40 μg/paw). The monoacylglycerol lipase (MAGL) inhibitor, JZL184 (4 μg/paw), and the dual MAGL/fatty acid amide hydrolase (FAAH) inhibitor, MAFP (0.5 μg/paw), potentiated the peripheral antinociceptive effect of low-dose kahweol. Furthermore, kahweol increased the levels of the endocannabinoid anandamide, but not of the other endocannabinoid 2-arachidonoylglycerol nor of anandamide-related *N*-acylethanolamines, in the plantar surface of the rat paw. Our results suggested that kahweol induced peripheral antinociception via anandamide release and activation of CB_1_ cannabinoid receptors and this compound could be used to develop new drugs for pain relief.

## Introduction

Kahweol is a diterpenoid that is found in the unsaponifiable lipid fraction of coffee seeds (1). The content of kahweol in a coffee drink depends on the preparation mode. Scandinavian, Turkish, and French press-style coffees contain high levels of kahweol, whereas filtered, percolated and instant coffees contain low levels of this diterpene (2). Kahweol has been shown to exhibit both adverse and protective biological effects. It is widely reported that this diterpene may increase the blood levels of cholesterol in both humans and animal models (3). However, kahweol has therapeutic actions that are primarily related to protective effects against the action of carcinogens (4). This natural compound may enhance the detoxification of carcinogens and mutagens via the induction of carcinogen-detoxifying enzyme systems, such as glutathione S-transferase and uridine 5'-diphospho-glucuronosyl-transferase (5).

Similar to the benefits observed in cancer therapy, kahweol has been shown not only to exhibit anti-inflammatory activity *in vivo* but also to inhibit the *in vitro* production of prostaglandin E_2_ (PGE_2_), a primary contributor to one of the key features of inflammation, namely, pain hypersensitivity (6,7). However, the mechanisms of the antinociceptive and anti-inflammatory activity of kahweol have not been well established. In this regard, our research group has described that kahweol-induced peripheral antinociception is accompanied by the release of endogenous opioid peptides and noradrenalin (8,9).

The opioid and noradrenergic systems are closely related to the endocannabinoid system (10,11). Our research group, for example, described the participation of endocannabinoids and CB_1_ cannabinoid receptors in central (12) and peripheral (13) antinociception induced by treatment with the μ-opioid agonist morphine. The CB_1_ cannabinoid and μ-opioid receptors are similarly distributed in the periaqueductal grey matter, gelatinous substance, (14,15) dorsal horn, dorsal root ganglion, and primary afferent neurons, regions that are closely related to central and peripheral antinociception (11,16,17), which provided an anatomical basis for our discovery. Additionally, the CB_1_ cannabinoid receptor antagonist SR141716A antagonizes the spinal antinociceptive effect of the adrenoceptor agonist clonidine (18).

Based on this background, since the opioid and noradrenergic systems were shown to be involved in the peripheral antinociception induced by kahweol, and considering the interactions between the opioid, noradrenergic, and endocannabinoid systems, the aim of the present study was to determine whether the endocannabinoid signaling system is involved in the peripheral antinociception induced by kahweol.

## Material and Methods

### Animals

All experiments were performed on 7-week-old male Wistar rats weighing 180-200 g obtained from the Bioterium Center of Federal University of Minas Gerais (CEBIO-ICB). The animals were housed in a temperature-controlled room (24±2°C) on an automatic 12-h light/dark cycle (06:00-18:00 h). All tests were conducted during the light phase. Food and water were freely available until the onset of the experiments. Following the experimental procedures, the animals were euthanized by an intraperitoneal injection of 10% ketamine hydrochloride (Dopalen, Vetbrands, Brazil) (180 mg/kg) plus 2% xylazine hydrochloride (Dopaser, Calier, Brazil) (15 mg/kg) in 100 µL saline. All animal procedures were conducted in accordance with the ethical guidelines of the International Association for the Study of Pain (IASP) (19) and had approval of the Committee of Ethics in Animal Experimentation of the Federal University of Minas Gerais (CETEA-UFMG; protocol No. 41/2007).

### Measurement of nociceptive threshold

Hyperalgesia was induced by subcutaneous injection of PGE_2_ (2 μg) into the plantar surface of the hind paw and was measured according to the paw pressure test as previously described (20). An analgesiometer (Ugo-Basile, Italy) with a cone-shaped paw-presser with a rounded tip was used, which applies a linearly increasing force to the hind paw. The weight in grams (g) required to evoke paw withdrawal (nociceptive response) was determined as the nociceptive threshold. A cutoff value of 300 g was used to reduce the risk of paw damage. The nociceptive threshold was measured in the right paw and determined as the average of the 3 consecutive trials recorded before and 180 min after PGE_2_ intraplantar injection. Hyperalgesia was calculated as the difference between these 2 averages (Δ of nociceptive threshold) and reported in grams.

### Drug administration

All drugs were administered by intraplantar injection of 50 μL/paw, except PGE_2_ (100 μL/paw). The tested natural product kahweol (C_20_H_26_O_3_; purity >98%; Axxora, USA) was dissolved in 10% dimethyl sulfoxide (DMSO). Monoacylglycerol lipase/fatty acid amide hydrolase inhibitor (MAFP) ([5*Z*,8*Z*,11*Z*,14*Z*]-5,8,11,14-eicosatetraenyl-methyl ester phosphonofluoridic acid; purity >98%; Tocris, USA) (0.5 µg/paw), an inhibitor of fatty acid amide hydrolase (FAAH), and monoacylglycerol lipase (MAGL) were dissolved in 3% ethanol. JZL184 (4-[Bis{1,3-benzodioxol-5-yl}hydroxymethyl]-1-piperidinecarboxylic acid 4-nitrophenyl ester; purity >98%; Tocris) (4 µg/paw), a selective inhibitor of MAGL, was dissolved in 20% DMSO. VDM11 ([5*Z*,8*Z*,11*Z*,14*Z*]-*N*-[4-hydroxy-2-methylphenyl]-5,8,11,14-eicosatetraenamide; purity >98%; Tocris) (2.5 µg/paw), a selective inhibitor of AEA cellular reuptake, was dissolved in 10% Tocrisolve. The CB_1_ cannabinoid receptor antagonist AM251 (*N*-[piperidin-1-yl]-5-[4-iodophenyl]-1-[2,4-dichlorophenyl]-4-methyl-1*H*-pyrazole-3-carboxamide; purity>99%; Tocris) (20, 40, 80 µg/paw) and the CB_2_ cannabinoid receptor antagonist AM630 (6-Iodo-2-methyl-1-[2-{4-morpholinyl}ethyl]-1H-indol-3-yl [4-ethoxyphenyl] methanone; purity >98%; Tocris) (100 µg/paw) were dissolved in 10% DMSO, whereas the hyperalgesic agent PGE_2_ (purity ≥93%; Sigma-Aldrich, USA) was dissolved in 2% ethanol.

### Experimental protocol

**Figure 1:**
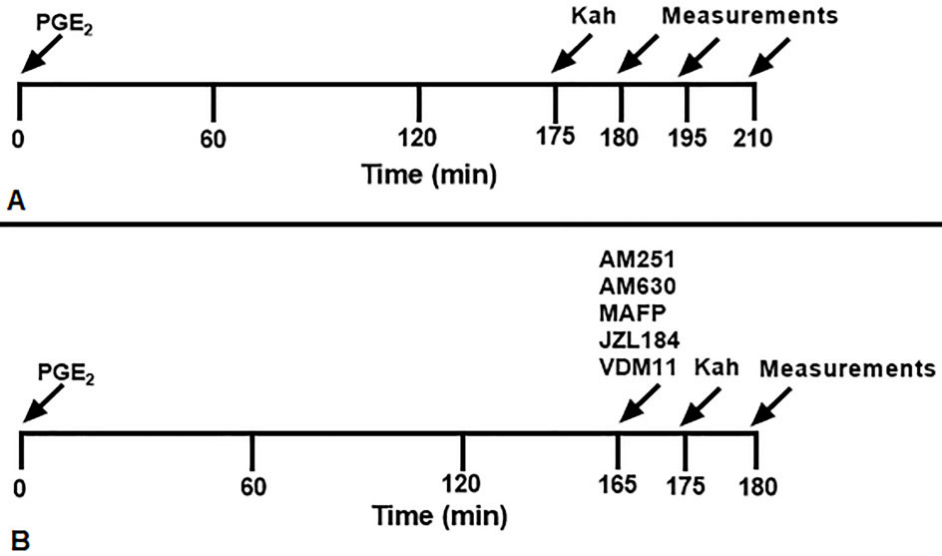
Schematic diagram of the experimental protocol. **A**, Kahweol (Kah) was administered 175 min after the local administration of prostaglandin E_2_(PGE_2_) (2 µg) and the antinociceptive response was measured prior to and 180 min, 195 min, and 210 min after PGE_2_ injection. **B**, Kah was administered in the right hind paw 175 min after local injection of PGE_2_. The cannabinoid drugs AM251, AM630, MAFP, JZL184, or VDM11 were given 10 min prior (165 min) to Kah intraplantar administration and measurements were performed prior to and 180 min after PGE_2_ administration.

To verify the temporal development of the effect induced by intraplantar administration of kahweol in hyperalgesia, PGE_2_ was administered at 0 min and kahweol was administered at 175 min, 5 min before the peak action of PGE_2_. Then, measurements were taken prior to and 180 min, 195 min and 210 min after PGE_2_ injection. Both the application of the two drugs and the measurements were performed in the right hind paw (Figure 1A).

To test the participation of the cannabinoid system in the analgesic action, kahweol was administered in the right hind paw 175 min after local injection of PGE_2_ and the measurements were performed prior to and 180 min after PGE_2_ administration. The cannabinoid drugs AM630, AM251, MAFP, JZL184, or VDM11 were given 10 min prior to kahweol intraplantar administration (165 min) (Figure 1B). The protocols concerning doses and time of injection of each drug used in this study were obtained through pilot experiments and literature data (13,21).

### Endocannabinoid extraction and quantification

Lyophilized lipid extracts from the hind paw skin were resuspended in chloroform/methanol 2:1 by volume. The solutions were then purified by openbed chromatography on silica as described in Bisogno et al. (22). Fractions eluted with chloroform/methanol 9:1 by volume (containing anandamide (AEA) and 2- arachidonoylglycerol (2-AG)) were collected, and the excess solvent evaporated with a rotating evaporator. In addition, aliquots were analyzed by isotope dilution-liquid chromatography (LC)/atmospheric pressure chemical ionization/mass spectrometry (MS), carried out under conditions described previously and allowing the separations of 2-AG and AEA. MS detection was carried out in the selected ion-monitoring mode using *m*/*z* values of 356 and 348 (molecular ions -1 for deuterated and undeuterated AEA), 384.35 and 379.35 (molecular ions -1 for deuterated and undeuterated 2-AG), 304 and 300 (molecular ions -1 for deuterated and undeuterated palmitoylethanolamide (PEA)), and 330 and 326 (molecular ions -1 for deuterated and undeuterated oleoylethanolamide (OEA)). The area ratios between signals of deuterated and undeuterated compounds varied linearly with varying amounts of undeuterated compounds (30 fmol to 100 pmol). Therefore, AEA, PEA, OEA, and 2-AG levels in unknown samples were calculated based on their area ratios with the internal deuterated standard signal areas.

### Statistical analysis

The data are reported as means±SE, and as all experiments had three or more groups, statistical comparisons among them were carried out using analysis of variance (ANOVA) followed by Bonferroni's test using Prism 6 (GraphPad Software, USA). Significance was accepted when P<0.05.

## Results

**Figure 2:**
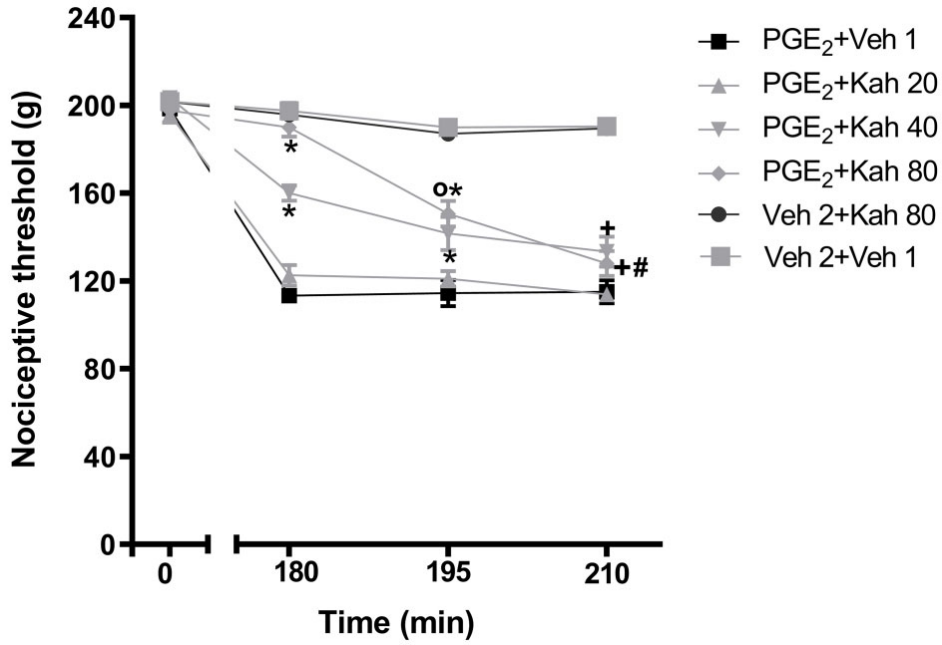
Temporal development of the effect induced by intraplantar administration of kahweol on hyperalgesia. Kah was administered 175 min after the local administration of prostaglandin E_2_(PGE_2_) (2 µg) and the antinociceptive response was measured prior to and 180 min, 195 min, and 210 min after PGE_2_ administration. Kah 20: kahweol 20 µg/paw; Kah 40: kahweol 40 µg/paw; Kah 80: kahweol 80 µg/paw; Veh (vehicle) 1: 2% ethanol in saline; Veh 2: 10% DMSO in saline. Data are reported as means±SE (n=5). *P<0.05 compared with PGE_2_+Veh 2. ^o^P<0.05, between the experimental periods 180 min and 195 min. ^#^P<0.05, between the experimental periods 195 min and 210 min. ^+^P<0.05, between the experimental periods 180 min and 210 min (ANOVA and Bonferroni's test).

A dose-dependent antinociceptive effect was observed with injection of kahweol (20, 40, and 80 µg/paw) into the right hind paw against PGE_2_-induced hyperalgesia (2 µg/paw). Kahweol (20 µg/paw) did not reach statistical significance, but 40 and 80 µg/paw were significant at 5 and 20 min after kahweol injection, and at 5 min, there was statistical significance between the doses of 40 and 80 µg/paw. The highest dose of kahweol (80 µg/paw) reached a complete reversal of the hyperalgesia in 5 min (Figure 2). Intraplantar injections of 2% ethanol, 10% DMSO solution, or kahweol alone did not have any antinociceptive or hyperalgesic effects [F (5,18)=2, P=0.0405].

**Figure 3:**
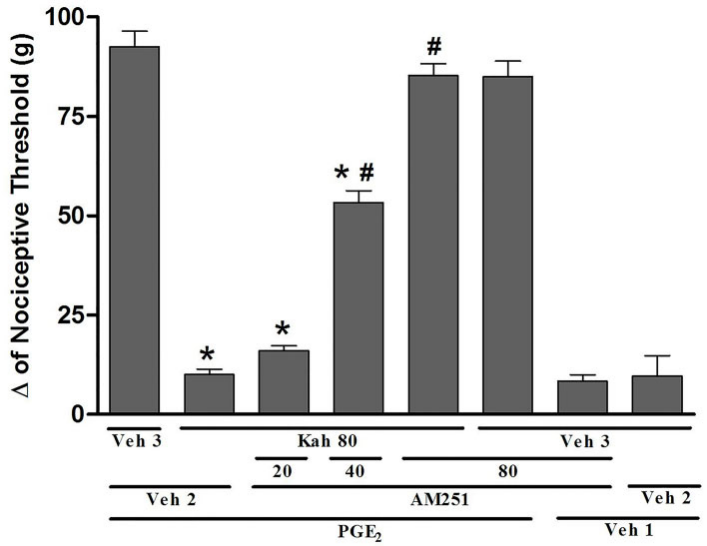
The CB_1_ receptor antagonist blocked kahweol-induced peripheral antinociception in hyperalgesic paws. The antinociceptive response was measured by the paw pressure test. Prostaglandin E_2_(PGE_2_) injection (2 µg/paw) was done at time 0, AM251 (20, 40, 80 µg/paw) was injected at time 165 min, and kahweol (Kah; 80 µg/paw) was given at 175 min. Measurements were made prior to and 180 min after PGE_2_ administration. Data are reported as means±SE (n=5) of Δ nociceptive threshold measured in grams (g). *P<0.05 compared to PGE_2_ + Veh 2 + Veh 3; ^#^P<0.05 compared to PGE_2_ + Veh 2 + Kah 80)-injected groups (ANOVA and Bonferroni's test). Veh (vehicle) 1: 2% ethanol in saline; Veh 2: 10% DMSO in saline; Veh 3: sterile saline solution (0.9% NaCl).

**Figure 4:**
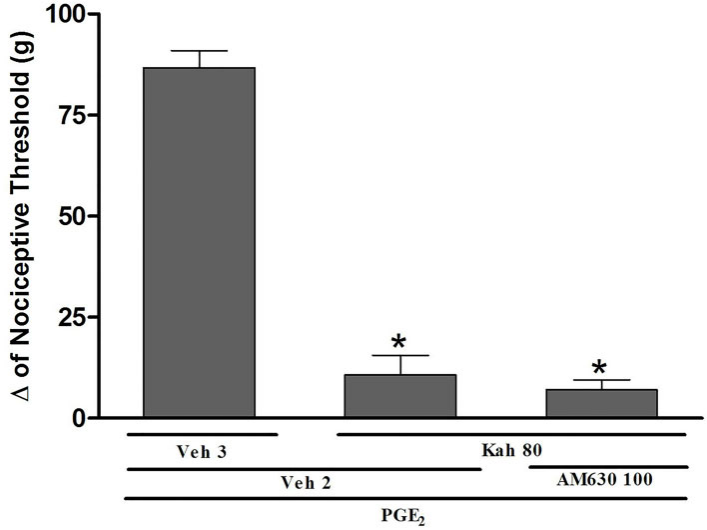
The CB_2_ receptor antagonist did not block kahweol-induced peripheral antinociception in hyperalgesic paws. The antinociceptive response was measured by the paw pressure test. Prostaglandin E_2_(PGE_2_) injection (2 µg/paw) was done at time 0, AM630 (100 µg/paw) was injected at time 165 min, and kahweol (Kah; 80 µg/paw) was given at 175 min. Measurements were made prior to and 180 min after PGE_2_ administration. Data are reported as means±SE (n=5) of Δ nociceptive threshold measured in grams (g). *P<0.05 compared to PGE_2_ + Veh 2 + Veh 3-injected group (ANOVA and Bonferroni's test). There was no significant difference between (PGE_2_ + Veh 2 + Kah 80) and (PGE_2_ + AM630 100 + Kah 80)-injected groups. Veh (vehicle) 2: 10% DMSO in saline; Veh 3: sterile saline solution (0.9% NaCl).

The effect of the highest dose of kahweol (80 μg/paw) was fully reverted by the selective CB_1_ cannabinoid receptor antagonist AM251 (20, 40, and 80 μg/paw) in a dose-dependent manner [F (97,27)=147.5, P=0.0001] (Figure 3). On the other hand, the intraplantar injection of the CB_2_ cannabinoid receptor antagonist AM630 (100 μg/paw) did not significantly reduce the antinociceptive effect of kahweol (80 μg/paw) [F (2,13)=130.8, P=0.0001] (Figure 4).

**Figure 5:**
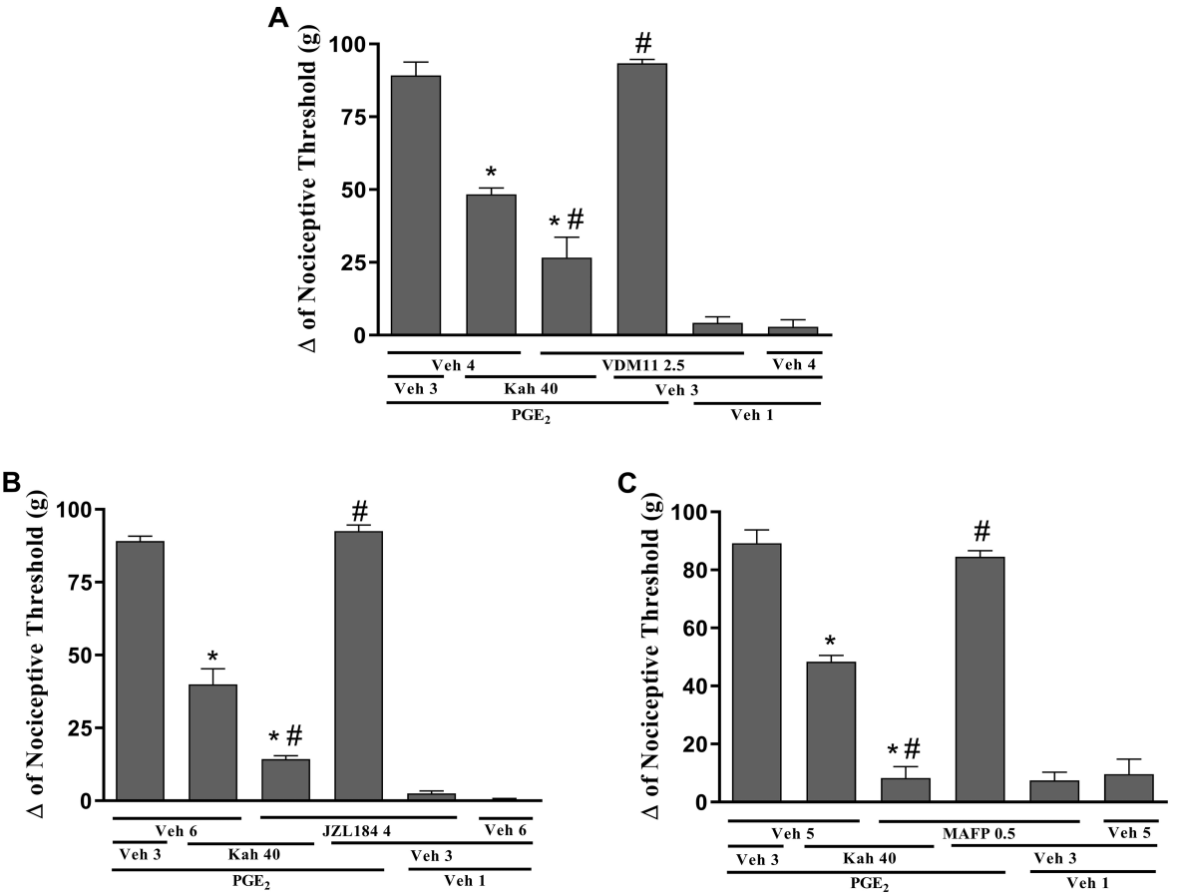
Pretreatment with VDM11, JZL184, and MAFP potentiated the peripheral antinociceptive action of kahweol. The antinociceptive response was measured by the paw pressure test. Prostaglandin E_2_(PGE_2_) injection (2 µg/paw) was done at time 0. VDM11 (2.5 µg/paw; **A**), JZL184 (4 µg/paw; **B**), or MAFP (0.5 µg/paw; **C**) was injected at time 165 min, and kahweol (Kah; 80 µg/paw) was given at 175 min. Measurements were made prior to and 180 min after PGE_2_ administration. Data are reported as means±SE (n=5) of Δ nociceptive threshold measured in grams (g). *P<0.05 compared to (PGE_2_ + Veh 3 + Veh 4/Veh 5/Veh 6); ^#^P<0.05 compared to (PGE_2_ + Kah 80 + Veh 4/Veh 5/Veh 6)-injected groups (ANOVA and Bonferroni's test). Veh (vehicle) 1: 2% ethanol in saline; Veh 3: sterile saline solution (0.9% NaCl); Veh 4: 10% tocrisolve in saline; Veh 5: 3% ethanol in saline; Veh 6: 20% DMSO in saline.

Once the participation of CB_1_ cannabinoid receptor in the mechanism of kahweol-induced antinociception was evaluated, the next step was to assess the endocannabinoid involvement in this process. The intraplantar injection of the endocannabinoid reuptake inhibitor, VDM11 (2.5 μg/paw) alone, did not induce antinociception, whereas when co-administered with low-dose kahweol (40 μg/paw), its antinociception was potentiated [F (5, 18)=111.9, P<0.0001] (Figure 5A). MAGL and FAAH inhibitors, JZL184 (selective for MAGL, 4 μg/paw) [F (5,18)=276.6, P<0.0001] and MAFP (active on both FAAH and MAGL, 0.5 μg/paw) [F (5,18)=111.1 P<0.0001], did not induce antinociception against PGE_2_-induced hyperalgesia by themselves, but enhanced the peripheral antinociceptive effect of low-dose kahweol (40 μg/paw) (Figure 5B and C).

**Figure 6:**
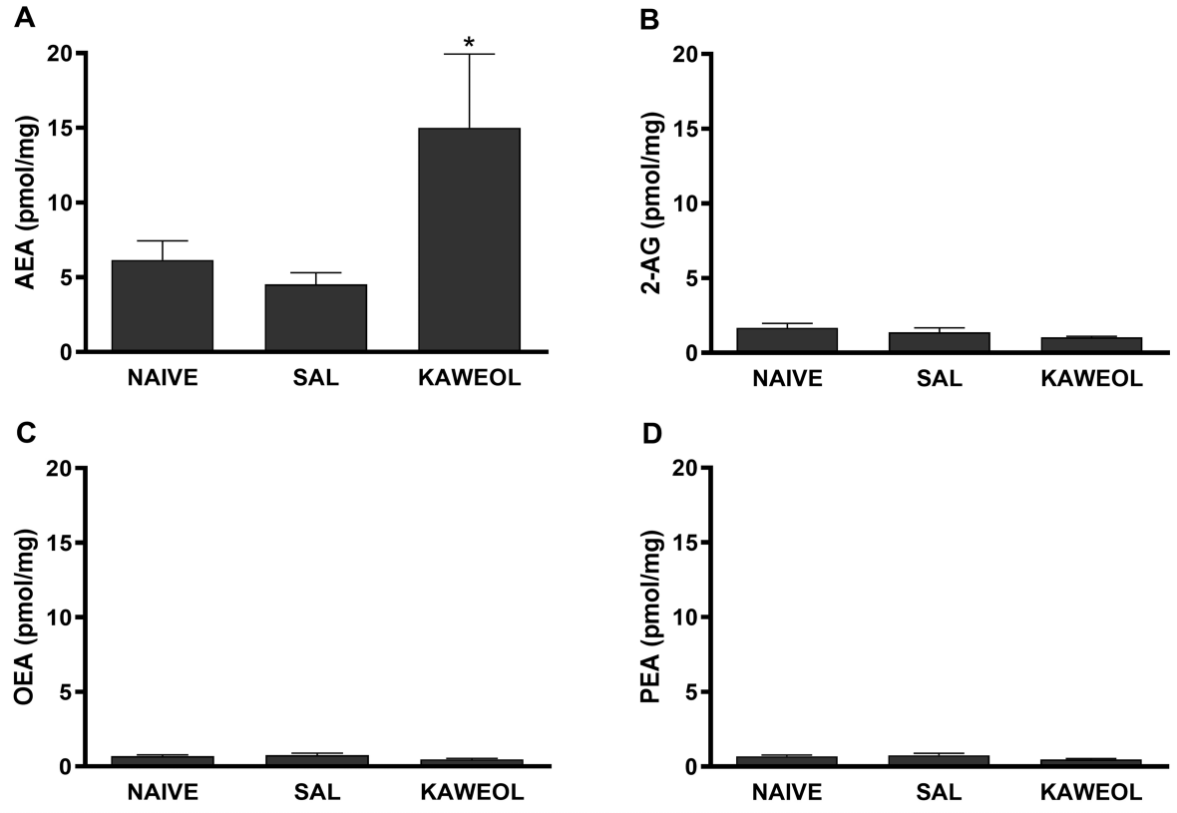
Increase of anandamide (AEA) levels (**A**), but not 2-arachidonoylglycerol (2-AG) (**B**), oleoylethanolamide (OEA) (**C**), and palmitoylethanolamide (PEA) (**D**) on kahweol injection. Kahweol (Kah; 80 μg/paw) was injected into the paw and 5 min later the paw tissues were collected for analysis. Data are reported as means±SE (n=5). *P<0.05 compared to Naive- and Saline (SAL)-injected groups (ANOVA and Bonferroni's test).

To determine which endocannabinoid could be involved in kahweol-induced peripheral antinociception, isotope dilution-LC-MS was used to quantify endocannabinoid levels in the lipid extracts of the rat paw tissue of animals that had been treated with kahweol. Treatment with kahweol induced a significant increase in AEA concentration [F (6,20)=3.675, P=0.0127] (Figure 6A). On the other hand, the same response was not observed for 2-AG [F (6,21)=5.047, P=0.0024], OEA [F (6,21)=2.357, P=0.0674], and PEA [F (6,21)=2.357, P=0.0674] The levels of these compounds remained unaltered in all groups (Figure 6B, C, and D).

## Discussion

Kahweol is a coffee-derived food compound with therapeutic properties, including antitumor and antinociceptive effects (5,8,9). Based on previous reports (8,9), in the present study, we used two different doses of kahweol, total and partial, for the induction of peripheral antinociception, against PGE_2_-induced hyperalgesia. The hyperalgesic agent PGE_2_ is a physiologically active lipid mediator that is able to sensitize primary afferent neurons to chemical, thermal, and mechanical stimuli (6). It was demonstrated that the intraplantar injection of PGE_2_ induced hyperalgesia in the rat paw compression test, with a peak at the 3rd hour after its administration (23).

With the purpose of evaluating the involvement of the endocannabinoid system in the peripheral antinociceptive effect of kahweol, we first did a temporal and dose response curve that demonstrated a similar curve to the cafestol curve (24). However, at the dose of 40 µg/paw, kahweol had a longer-lasting antinociceptive effect than cafestol. It is important to note that control solutions (ethanol and DMSO) in concentrations used in this research did not cause any antinociceptive or hyperalgesic effects, as demonstrated in previous studies (25).

By using intraplantar injection, it is possible to deliver small quantities of drugs directly to this compartment. In the present study, the peak of the antinociceptive effect of kahweol was observed at 3 h, and in the following 30 min this effect decreased, as the intraplantar administration undergoes absorption kinetics.

It is well described that endogenous and exogenous cannabinoid receptor agonists may produce antinociception by receptor activation and that this process might be reversed by the use of pharmacological antagonists (26). A previous report from our research group showed that the peripheral antinociceptive effects of AEA, an endogenous CB_1_ cannabinoid agonist, and PEA, an AEA-related lipid mediator, which might indirectly activate CB_2_ receptors, were blocked by the CB_1_ and CB_2_ receptor antagonists, AM251 and AM630, respectively, in a dose-dependent manner (21). Therefore, in the present study, we used these pharmacological tools in order to achieve our purposes.

The CB_1_ cannabinoid receptor antagonist AM251 antagonized the peripheral effect of kahweol in a dose-dependent manner, suggesting the involvement of this receptor in kahweol-induced peripheral antinociception. These receptors are densely expressed in the superficial lamina of the dorsal horn of the spinal cord, in the dorsal root ganglion, and in the peripheral terminals of primary afferent neurons (27), thus providing anatomical support to the reversal of the kahweol-induced antinociceptive effect by AM251. In addition, in previous studies, treatment with AM251 also abolished the antinociceptive action of acetaminophen (28). On the other hand, the involvement of the CB_2_ cannabinoid receptor in the peripheral antinociception induced by kahweol was ruled out here since the intraplantar administration of the CB_2_ cannabinoid receptor antagonist AM630 did not antagonize the antinociceptive effect of kahweol. Importantly, AM630, at the same dose and in the same experimental model used in this study, was previously found to antagonize the peripheral antinociception induced by PEA (21).

Once we confirmed the involvement of the CB_1_ receptor in the kahweol-induced peripheral antinociceptive effect, we then evaluated the involvement of endocannabinoids in this mechanism. Endocannabinoid cellular reuptake inhibitors are useful in the manipulation of endocannabinoid levels under both physiological and pathological conditions (29). Despite the fact that the existence of a plasma membrane transporter for endocannabinoid cellular reuptake is still controversial, it is well established that part of the endocannabinoid action is terminated through this latter process (30). The intraplantar injection of the endocannabinoid reuptake inhibitor VDM11 was able to potentiate the antinociception of kahweol, suggesting the involvement of endocannabinoids in this mechanism.

Besides the involvement of the transporter, the endocannabinoid signaling is also limited by the action of intracellular serine hydrolases, such as MAGL and FAAH. FAAH is mainly involved in AEA degradation, with the subsequent release of ethanolamine and arachidonic acid as end products. On the other hand, MAGL is primarily involved in the degradation of 2-AG, which results in the release of arachidonic acid and glycerol as final products (31,32). Therefore, the treatment with FAAH and MAGL inhibitors may increase AEA and 2-AG levels, respectively, in nervous tissue which, at least in part, explains their antinociceptive activity in rodents (33,34). Furthermore, it has been shown that these inhibitors, when administered peripherally, may enhance the peripheral antinociceptive effects that are induced by low doses of endocannabinoid agonists (34). Here, it was shown that MAGL and FAAH inhibitors, JZL184 and MAFP respectively, did not induce antinociception against PGE_2_-induced hyperalgesia, but enhanced the peripheral antinociceptive effect of low-dose kahweol. Endocannabinoids are released by demand by some cells such as keratinocytes and several immune cells that not only can be influenced by these bioactive lipids, but can also generate and release them (35,36). Therefore, a change was observed in nociception with the inhibitors without kahweol and there was a potentiation when kahweol was added. These results suggested that the intraplantar injection of kahweol might induce release of endocannabinoids that may act predominantly via CB_1_ receptors.

To analyze the concentrations of the endocannabinoids AEA and 2-AG, as well as of the AEA-related compounds, PEA and OEA, in paw tissues after kahweol treatment, isotope-dilution LC-MS analyses of the paw lipid extracts was used. These measurements showed a significant increase in AEA levels in the paws of kahweol-treated animals. Thus, AEA appears to be mobilized by kahweol, which is in agreement with the selective sensitivity to the CB_1_ receptor antagonist of kahweol antinociceptive effect, observed in this study; in fact, AEA is more efficacious at the CB_1_ than CB_2_ cannabinoid receptors (26).

The levels of 2-AG, an endocannabinoid with equal efficacy at CB_1_ and CB_2_ receptors (26), were not increased in the presence of kahweol, which suggested that this mediator was not involved in kahweol antinociception. In addition, the levels of OEA and PEA, which do not directly activate CB_1_ or CB_2_ receptors and act preferentially via other targets (28), were not increased by kahweol as well.

Many hypotheses could be made about the mechanism by which kahweol releases anandamide in the periphery. In a previous study, our research group demonstrated for the first time that kahweol induces a release of endogenous opioid peptides, possibly through activation of µ-opioid receptors in nociceptive afferent neurons resulting in an antinociceptive action (8). In addition, the opioid system activation has been linked to endocannabinoid release. The antinociceptive effect of a μ-opioid receptor agonist, but not δ and κ agonists, was blocked by the administration of a CB_1_ cannabinoid receptor antagonist peripherally and centrally, suggesting that the central and peripheral antinociceptive effect of morphine may be mediated by the endocannabinoid system (12,13). Thus, we suggest that kahweol-induced µ-opioid receptor activation may trigger AEA release, leading to the activation of the CB_1_ cannabinoid receptor.

Another hypothesis is based on the fact that noradrenalin may also be involved in kahweol-induced peripheral antinociception (9). The activation of α_1_, α_2_, and β_2_ adrenoceptors in immune cells during the inflammatory process may release endogenous opioids (37). These receptors increase the cellular levels of cAMP, induce PKA activation, and subsequently increase intracellular calcium (38), a mechanism involved in endocannabinoid release (39). Furthermore, a synergic antinociceptive effect between the α_2_ adrenoceptor agonist clonidine and the CB_1_ cannabinoid agonist WIN 55,212-2 was demonstrated in the spinal cord (40). Therefore, it is possible that kahweol releases noradrenaline and indirectly activates adrenoceptors, thereby inducing an increase in intracellular calcium with subsequent AEA release, CB_1_ activation, and antinociception.

In conclusion, our results contribute to the understanding of the mechanisms underlying the antinociception induced by some food compounds, indicating that the coffee-derived kahweol induced peripheral CB_1_ cannabinoid receptor activation with the involvement of the endocannabinoid AEA in this pharmacological action, suggesting a therapeutic potential of this natural product as an antinociceptive drug.
